# A Screen of Plant-Based Natural Products Revealed That Quercetin Prevents Pyroglutamylated Amyloid-β (Aβ3(pE)-42) Uptake in Astrocytes As Well As Resulting Astrogliosis and Synaptic Dysfunction

**DOI:** 10.1007/s12035-024-04509-6

**Published:** 2024-09-25

**Authors:** Helene Arndt, Mark Bachurski, PingAn Yuanxiang, Katrin Franke, Ludger A. Wessjohann, Michael R. Kreutz, Katarzyna M. Grochowska

**Affiliations:** 1 Research Group Neuroplasticity, Leibniz Institute for Neurobiology, 39118 Magdeburg, Germany; 2 Department of Bioorganic Chemistry, Leibniz Institute of Plant Biochemistry, 06108 Halle, Germany; 3 Institute of Biology/Geobotany and Botanical Garden, Martin Luther University Halle-Wittenberg, 06108 Halle, Germany; 4 German Centre for Integrative Biodiversity Research (iDiv) Halle-Jena-Leipzig, 04103 Leipzig, Germany; 5 Institut Für Chemie, Chair of Natural Products Chemistry, Martin-Luther-University Halle-Wittenberg, 06120 Halle (Saale), Germany; 6 Leibniz Group ‘Dendritic Organelles and Synaptic Function’, Center for Molecular Neurobiology, ZMNH, University Medical Center Hamburg-Eppendorf, 20251 Hamburg, Germany; 7 German Center for Neurodegenerative Diseases (DZNE), 39120 Magdeburg, Germany; 8 Center for Behavioral Brain Sciences, Otto Von Guericke University, 39120 Magdeburg, Germany

**Keywords:** Astrocytes, Alzheimer’s disease, Amyloid-β, Quercetin, Synaptic plasticity

## Abstract

Two connected histopathological hallmarks of Alzheimer’s disease (AD) are chronic neuroinflammation and synaptic dysfunction. The accumulation of the most prevalent posttranslationally modified form of Aβ1–42, pyroglutamylated amyloid-β (Aβ3(pE)-42) in astrocytes is directly linked to glial activation and the release of proinflammatory cytokines that in turn contribute to early synaptic dysfunction in AD. At present, the mechanisms of Aβ3(pE)-42 uptake to astrocytes are unknown and pharmacological interventions that interfere with this process are not available. Here we developed a simple screening assay to identify substances from a plant extract library that prevent astroglial Aβ3(pE)-42 uptake. We first show that this approach yields valid and reproducible results. Second, we show endocytosis of Aβ3(pE)-42 oligomers by astrocytes and that quercetin, a plant flavonol, is effective to specifically block astrocytic buildup of oligomeric Aβ3(pE)-42. Importantly, quercetin does not induce a general impairment of endocytosis. However, it efficiently protects against early synaptic dysfunction following exogenous Aβ3(pE)-42 application.

**Supplementary Information:**

The online version contains supplementary material available at 10.1007/s12035-024-04509-6.

## Introduction

Alzheimer’s disease (AD) is a devastating neurodegenerative condition with a poor prognosis. AD is the most common type of dementia and the histopathological hallmark of the disease is the accumulation of hyperphosphorylated tau tangles and extracellular plaques of amyloid-β (Aβ) [1, 2]. According to the most prominent hypothesis for causation of AD, the amyloid-hypothesis, the oligomerization and deposition of the Aβ peptide as fibrils in the brain is causative for disease progression [1–5]. Next to their direct neurotoxic properties, Aβ oligomers activate microglia and astrocytes, which in turn results in chronic sterile neuroinflammation and production of pro-inflammatory cytokines that are synaptotoxic [6–8]. Importantly, neuroinflammation is part of early disease pathology and is believed to contribute to late onset AD (LOAD) [1, 6]. Neuroinflammation in AD is orchestrated by microglia and astrocytes [6, 9]. Aβ can directly induce microglial immune response by binding to their receptors (e.g., Toll-like receptors or CD63) triggering the release of proinflammatory cytokines and microglia-driven synaptic pruning [6, 9]. Moreover, Aβ elicits astrocytic reactivity, leading to the release of synaptotoxic cytokines [6]. In addition, at early AD stages, astrocytes display disrupted homeostatic functions, especially calcium and glutamate dyshomeostasis, which contribute to synapse loss [10–12].

Multiple posttranslationally modified Aβ peptides were reported in AD patients and among those, the amino-terminally truncated, pyroglutamylated form of Aβ (Aβ3(pE)-42) is abundant in AD brains and seeds highly toxic co-oligomers with conventional Aβ1–42 [13–15]. So far, drug development was directed against various steps of these processes, e.g., against pyroglutamate formation [16, 17]. In previous work, we found that in contrast to Aβ1–42, Aβ3(pE)-42 is readily taken up by astrocytes, accumulates in the astrocytic endolysosomal system, leading to the lysosomal membrane permeabilization [18, 19]. The uptake of Aβ3(pE)-42 potently induces reactive astrogliosis and subsequent release of the proinflammatory cytokine TNFα, which results in synapse loss [18, 19]. Several lines of evidence support the notion that the synaptotoxic effects of N-terminally modified Aβ species are associated with glial activation [15, 19, 20]. It would therefore be beneficial to identify well-tolerated compounds that prevent the uptake of Aβ3(pE)-42 to astrocytes.

Plant-based natural products have a long history in medical applications although their full potential is still not appreciated [17]. In addition to their direct action, plant components can prevent disease or support treatment [21]. If they derive from food sources, they are well tolerated and allow rapid application, e.g., as nutraceutical with high patient acceptance and compliance and easy legal entrance to the market [22]. This includes plant natural products for the prevention or treatment of AD [23–26].

In the present study, we aimed to establish a screening assay that would allow to identify substances from plant libraries which can attenuate the reactive astrogliosis caused by Aβ3(pE)-42. In a second step, we then verified that one of the identified substances in this regard has indeed neuroprotective effects.

## Materials and Methods

### Aβ3(pE)-42 Oligomer Preparation

The Aβ3(pE)-42 peptide was ordered from AnaSpec (Cat. No. AS-29907). Aβ3(pE)-42 oligomers were prepared as described previously [19]. The quality of the production and procedure was controlled by SDS-PAGE (Fig. S1).

### SDS-PAGE and Western Blot

For verification of Aβ3(pE)-42 oligomerization, standard SDS-PAGE protocol followed by Western blot was used. The samples were mixed with 4 × SDS—sample buffer (250 mM Tris–HCl, pH 6.8, 1% (w/v) SDS, 40% (v/v) glycerol 20% (v/v) β-mercaptoethanol, 0.004% Bromophenol Blue) and loaded onto 5–20% polyacrylamide gel. The gel was set under a constant electric field of 12 mA for 1.5 h followed by transfer to a nitrocellulose membrane (Merck, Cat. No. GE10600002). The membranes were probed with anti-pE3 antibody (1:1000, SySy, Cat. No. 218003, RRID:AB_2056424) detected with anti-rabbit secondary antibody conjugated with HRP (Dianova Cat. No. 111–035-144, RRID:AB_2337938).

### Plant-Based Natural Products

Plant substances were selected from the Natural Product Library of the Leibniz-Institute of Plant Biochemistry, Dept. Bioorganic Chemistry, Halle (Saale). The substances were dissolved in dimethyl sulfoxide (DMSO, > 99%, Duchefa Biochemie) at a concentration of 10 mM. For treatment of astrocytes, the substances were further diluted in water with 0.5% DMSO to a concentration of 1 mM and subsequently applied to the culture medium resulting in a final concentration of 5 µM and 50 µM (max. DMSO content of 0.5%). The structures of the natural products are shown in Fig. S2.

### Primary Astrocyte Cell Culture

All animal experiments were carried out in accordance with the European Communities Council Directive (2010/63/EU) and German animal welfare act. Astrocytes were isolated from new-born rat forebrain tissue (P0) and stored at − 80 °C in Dulbecco’s Modified Eagle’s Medium (DMEM) (Gibco, Cat. No. 41966–029) following previously described procedures [19]. The cells were thawed in a water bath on demand and added to pre-warmed DMEM supplemented with 10% FBS, 1% penicillin/streptomycin, and 0.8 mM L-glutamine. Astrocytes were plated in 24-well plates with poly-D-lysine (Sigma-Aldrich, Cat. No. P-1149)-coated coverslips and kept at 37 °C, 5% CO2, and 95% humidity in a Heraeus incubator. After 24 h, the medium was exchanged to serum-free DMEM containing 1% penicillin/streptomycin.

### Screening Assay

Thirty-two hours following medium exchange, astrocytes were treated with the plant natural products for 16 h at a concentration of 5 µM or 50 µM and 500 nM Aβ3(pE)-42. Subsequently, cells were fixed in 4% PFA and 4% sucrose and permeabilized with 0.2% TX-100 in PBS for 10 min, blocked for 1 h in blocking solution (2% glycine, 0.2% gelatine, 2% BSA, and 50 mM NH_4_Cl (pH 7.4)), and incubated overnight at 4 °C with GFAP (1:500, SySy, Cat. No. 173–004, RRID:AB_10641162) and Aβ3(pE)-42 (1:500, SySy, Cat. No. 218003, RRID:AB_2056424) antibodies diluted in blocking buffer. Next, the samples were washed with PBS and incubated 2 h at RT with secondary antibodies: anti-guinea pig-AlexaFluor 488 (1:500, Thermo Fisher Scientific, Cat. No. A-11073, RRID:AB_2534117) and anti-rabbit-AlexaFluor 568 (1:500, Thermo Fisher Scientific, Cat. No. A-11036, RRID:AB_10563566) diluted in blocking buffer. Finally, the coverslips were mounted with Mowiol 4–88 (Merck Chemicals). Each condition was performed in duplicates. As controls, two coverslips were incubated with 500 nM Aβ3(pE)-42 only or vehicle control (0.5% DMSO, ITW Reagents, Cat. No. A3672).

Images were acquired from five randomly chosen field of views (FOV) per coverslip from two independent experiments, resulting in 20 FOVs per group, using a Zeiss Axio Imager A2 fluorescent microscope with 20 × objective (Zeiss, Plan-Apochromat, 20x/0,8 M27, Item No.: 420650–9901-000) equipped with the Cool Snap EZ Monochrome camera (Photometrics) and VisiView Imaging software (Visitron Systems) and analyzed with Image-J software [27]. For quantification of intracellular Aβ3(pE)-42 uptake, fixed threshold values were applied to both 16-bit channels (GFAP threshold at 135, Aβ3(pE)-42 at 1000). The following parameters were quantified: (1) area—the sum of areas from all detected Aβ3(pE)-42 deposits, (2) the number of deposits—the total number of Aβ3(pE)-42 deposits identified, (3) mean size—the average area from all Aβ3(pE)-42 deposits identified within one FOV. Aβ3(pE)-42 area and number of deposits were normalized to the respective GFAP area to account for the number of astrocytes within FOV and astrocytic size. Plotted values were normalized to the corresponding control. Since extracellular Aβ3(pE)-42 deposits were not observed, all signal from the Aβ3(pE)-42 channel was considered intracellular Aβ3(pE)-42. All experimental steps were performed blinded to the plant substances.

For endocytosis inhibition experiments, Aβ3(pE)-42 was co-applied with dynasore (Merck, Cat. No. 324410) at the final concentration of 5 µM for 16 h and analyzed in the same way as described above.

### HEK-293T Cell Culture

HEK-293T (ATCC, Cat. No. CRL-3216) cells were cultured in DMEM (Gibco, Cat. No. 41966–029) supplemented with 10% FBS, 1% penicillin/streptomycin, and 0.8 mM L-glutamine in an incubator at 37 °C, 5% CO2, and 95% humidity. Cells were plated in 24-well plates with coverslips coated with poly-D-lysine; 500 nM Aβ3(pE)-42 oligomers and 5 µM or 50 µM quercetin were added simultaneously after 10 h. Each condition was performed in duplicates. Following 16 h of incubation, cells were fixed in 4% PFA and 4% sucrose and stained according to the protocol described above with vimentin (1:200, Sigma-Aldrich, Cat. No. V-6630, RRID:AB_477627) and Aβ3(pE)-42 (1:500, SySy, Cat. No. 218003, RRID:AB_2056424) antibodies with secondary antibodies anti-mouse-AlexaFluor 488 (1:500, Thermo Fisher Scientific, Cat. No. A-11001, RRID:AB_2534069) and anti-rabbit-AlexaFluor 568 (1:500, Thermo Fisher Scientific, Cat. No. A-11036, RRID:AB_10563566). Image acquisition and analysis were done like described above.

### Mixed Primary Hippocampal Cultures

Rat hippocampi were dissected from E18-day-old Sprague Dawley rats and primary mixed astrocytic-neuronal cultures were prepared by technical assistants following a previously described protocol [19]. Cells were seeded in poly-D-lysine-coated 18-mm coverslips in 12-well plates at a density of 30,000 cells per coverslip. Neurons were kept in Neurobasal medium (Gibco, Cat. No. 21103049) supplemented with 0.8 mM glutamine (Gibco, Cat. No. 25030–024), 1% penicillin/streptomycin (Gibco, Cat. No. 15240–062), and 2% B27 (Gibco, Cat. No. 17504–044). Cultures were kept in the incubator at 37 °C, 5% CO2, and 95% humidity.

### Nuclear pCREB and Synaptic Staining, Imaging, and Quantification

On DIV, 16–18 mixed hippocampal cultures were incubated with 50 µM quercetin or 50 µM aristolactam BII and/or 500 nM Aβ3(pE)-42 oligomers for 2 (synaptic density assay) or 3 days (CREB shutoff assay), fixed with 4% PFA and 4% sucrose, and stained according to the protocol described above with antibodies detecting pCREB (Ser133) (1:500, Cell Signaling, Cat. No. 9198, RRID:AB_2561044) and MAP2 (1:500, Sigma-Aldrich; Cat. No. M-4403, RRID:AB_477193), detected with secondary antibodies anti-mouse-AlexaFluor 488 (1:500, ThermoFisher Scientific, Cat. No. A-11001, RRID:AB_2534069) and anti-rabbit-AlexaFluor 568 (1:500, ThermoFisher Scientific, Cat. No. A-11036, RRID:AB_10563566). Next, the samples were washed and co-stained with DAPI. For synaptic staining, the samples were stained with anti-PSD95 minibody (1:500, NanoTag N3783), anti-synaptophysin 1 (1:500, SySy, Cat. No. 101011, RRID:AB_887824), and anti-MAP2 (1:500, Sysy, Cat. No. 188004, RRID:AB_2138181) detected with anti-guinea pig-AlexaFluor 568 (1:500, ThermoFisher Scientific, Cat. No. A-11075, RRID:AB_2534119), anti-rabbit-AlexaFluor 488 (1:500, ThermoFisher Scientific, Cat. No. A-11034, RRID:AB_2576217), and anti-mouse-AlexaFluor 647 (1:500, Thermo Fisher Scientific, Cat. No. A-32733, RRID:AB_2633282).

Images were acquired with an inverse Leica TCS STED-SP8 3 × microscope (Leica-Microsystems, Germany) equipped with pulsed White Light Laser (WLL) and diode 405 nm for excitation with 63 × (Leica) objective lens along the *z*-axis with 300-nm *z*-step in a 512 × 512-pixel formats at 8-bit image depth at 400-Hz laser frequency.

Images were analyzed with ImageJ [27]. Nuclear pCREB intensities were measured within region of interest defined by DAPI. Synapses (overlap or opposing pre- and post-synaptic signal) were counted along defined dendritic stretches.

### Dextran Uptake Assay

To monitor endocytosis, 10-kDa fixable tetramethylrhodamine (TMR) dextran (5 mM, ThermoFisher Scientific, Cat. No. D1868) was applied to the cells together with 500 nM of Aβ3(pE)-42, 50 µM of quercetin, or Aβ3(pE)-42 with quercetin. Following 2-h pulse, the cells were kept 2 h in growth medium, and subsequently washed and fixed. The samples were next co-stained with antibodies detecting GFAP and Aβ3(pE)-42 as described above. The images were acquired with Olympus FV3000 confocal microscope (Evident, Germany) equipped with solid state laser lines 405 nm, 488 nm, and 561 nm for excitation with 60 × OlanApo Oil objective lens (NA: 1.4) along the *z*-axis with 300-nm *z*-step in a 512 × 512-pixel formats at 8-bit image depth. Averaged fluorescent intensity from four planes across optical axis was analyzed. Images were analyzed with ImageJ [27]. Dextran puncta were considered endocytosed if they appeared within GFAP region of the cell. The fluorescence maxima detection function was used to detect discrete dextran or Aβ3(pE)-42 puncta (circular region of interests (ROIs) with 3 pixels radius). For quantification of endocytosis rate, the total number of discrete puncta was normalized to GFAP area to account for size of astrocytic cell. The overlap between dextran and Aβ3(pE)-42 ROIs was considered as colocalization. Line profiles depict fluorescence intensity along the straight line normalized individually for each channel. For the representative images, the background subtraction and median filter were applied equally between groups.

### Organotypic Slice Cultures

Rat, organotypic hippocampal slices were prepared as described previously (Gee et al., 2017). Experiments were performed with cultures at DIV8 or 16. 500 nM Aβ3(pE)-42 and 50 μM quercetin were applied for 24 h. Slices were stained according to the above-described protocol, with the modified permeabilization step—0.3% TX-100 for 1 h at RT. The astrocytes were labeled with GFAP (1:500, SySy, Cat. No. 173–004, RRID:AB_10641162) or vimentin antibody (1:500, Agilent, Cat. No. M0725, RRID:AB 10013485) detected with anti-guinea pig-AlexaFluor 488 (1:500, ThermoFisher Scientific, Cat. No. A-11073, RRID:AB_2534117) or anti-mouse AlexaFluor 488 (1:500, ThermoFisher Scientific, Cat. No. A-11001, RRID:AB 2534069) and subsequently counterstained with DAPI. The images of samples were acquired with a Zeiss LSM 900 (Plan-Apochromat 20 × /0.8). Ten images from *z*-optical plane were acquired with a step size of 0.5 μm for each region of interest. Excitation/emission filters were selected using the dye selection function of the Fluoview/Zen3.5 software (Alexa 405 (DAPI), Alexa 488 (GFAP or vimentin)). The maximum fluorescence intensity was quantified using ImageJ [27] excluding pyramidal layer region defined by DAPI. Images were not deconvolved or filtered for quantification. Representative images presented in the figures were linearly adjusted.

### Acute Hippocampal Slice Preparation and Long-Term Potentiation (LTP) Recording

Acute hippocampal slices from 8- to 12-week-old male C57/B6J mice were prepared according to a previously described protocol [28]. Briefly, mouse brains were sliced by Vibratome (Leica VT1000ST) into 350-μm-thick slices. Hippocampal slices were incubated with 500 nM Aβ3(pE)-42 and 10 µM quercetin in carbogenated (CO2 5%, O2 95%) aCSF solution (110 mM NaCl, 2.5 mM KCl, 2.5 mM CaCl2, 1.5 mM MgSO4, 1.24 mM KH2PO4, 10 mM glucose, 27.4 mM NaHCO3, pH 7.3) at room temperature for 2 h. Field excitatory postsynaptic potentials (fEPSPs) were measured in stratum radiatum after stimulation of CA1 Schaffer-collateral fibers, amplified by Extracellular Amplifier (EXT-02B, npi, Germany), and digitated at a sample frequency of 5 kHz by Digidata 1201 plus AD/Da converter (CED, UK). To induce LTP tetanization with 3 × 1 s stimulus trains at 100 Hz with a 10-min inter-train interval and double-pulse width (0.2 ms) were used.

### Statistical Analysis and Figure Preparation

GraphPad Prism was used to plot graphs and perform statistical analyses. For the screening assay, outliers were removed using ROUT method, with *Q* = 1% or Grubb’s outlier test *α* = 0.05 for synapse density, pCREB, and GFAP intensity quantification. The statistical test was chosen based on the normal distribution of the data sets determined by D’Agostino-Pearson test. For normally distributed data, two-way ANOVA with Tukey post hoc test for multiple comparisons was used. For not normally distributed data, Kruskal–Wallis followed by Dunn’s multiple comparisons test was used. For HEK293-T-cell experiments, one-way ANOVA was performed followed by Dunnett’s test for multiple comparisons. Results from statistical tests are indicated within each figure legend; statistical notations in the graphs are as follows: ns, nonsignificant; *p ≤ 0.05; **p ≤ 0.01; ***p ≤ 0.001; ****p ≤ 0.0001. All figures were prepared with Adobe Illustrator.

## Results

**Fig. 1:**
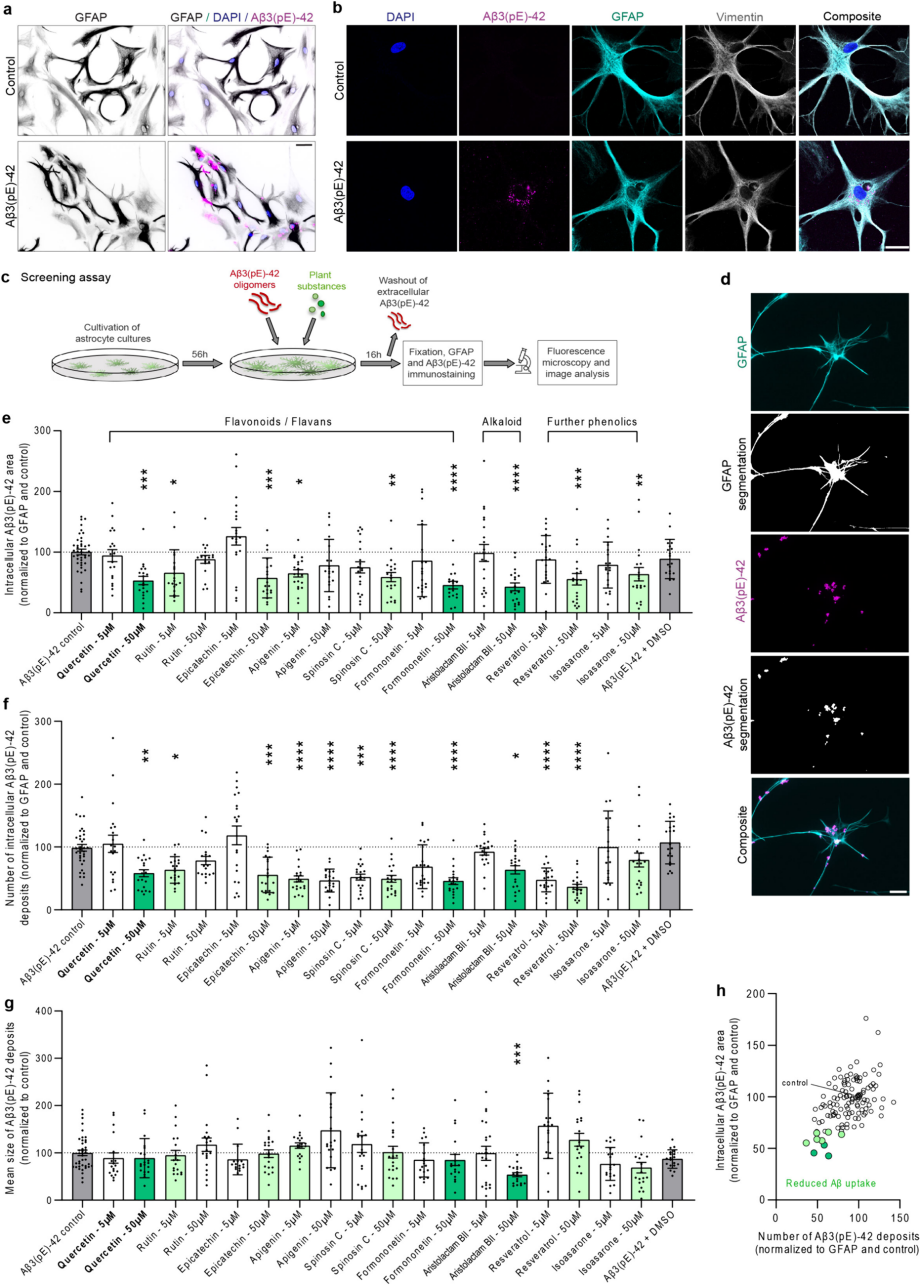
Plant substances alter uptake and morphology of astrocytic, intracellular Aβ3(pE)-42 deposits. **a**, **b** Astrocytes take up Aβ3(pE)-42 oligomers in vitro, while no extracellular deposits are visible. Representative **a** fluorescence and **b** confocal images of astrocytes. Scale bar **a** 50 µm and **b** 25 µm. **c** Schematic workflow of the screening assay. **d** Segmentation of the GFAP and Aβ3(pE)-42 fluorescent signal used for analysis. Scale bar 50 µm. **e**–**g** Selected plant substances lead to altered **e** intracellular area, **f** number, and **g** size of intracellular Aβ3(pE)-42 deposits. Plant substances highlighted in green reduce intracellular Aβ3(pE)-42 area and number of deposits. Quercetin (50 μM), formononetin (50 µM), and aristolactam BII (50 µM) are marked in dark green. Twenty fields of view (FOVs) per condition from two independent experiments were analyzed. Data were normalized to the corresponding control and for **e**, **f** also to GFAP area per FOV. Control represents incubation with Aβ3(pE)-42 only; Aβ3(pE)-42 plus vehicle control for plant substances (DMSO) showed no effect (both in grey). **h** Number of Aβ3(pE)-42 deposits versus intracellular Aβ3(pE)-42 area was plotted for all tested plant substances. The same nine plant substances like in **e**–**g** are highlighted in green and dark green. **e**–**g** $$*$$ *p* < 0.05; $$**$$ *p* < 0.01; $$***$$ *p* < 0.001; $$****$$ *p* < 0.0001 by Kruskal–Wallis test followed by Dunn’s multiple comparisons test. Data are presented as mean $$\pm$$ s.e.m

Aβ accumulation, oligomerization, and deposition are central to the pathogenesis of AD [1–3]. Here we took advantage of a Natural Product library from the Leibniz Institute of Plant Biochemistry to search for substances that directly target the uptake of Aβ3(pE)-42 oligomers in astroglia. We initially screened 61 substances that were pre-selected from the 30,000 entries library based on the following criteria: (1) availability/amounts accessible; (2) chemical stability; (3) small molecule, ideally obeying Lipinsky rules; (4) natural product (NP) or simple NP derivative, preferentially from plant or mushroom; (5) derived from a plant with reported anti-Alzheimer or similar neurological activity, including such data from our previous research; or: from a compound class reported beneficial to neuro-inflammation; or: compound with structural similarity to such with reported activity in mental health [16, 25, 26, 29–32]. The tested natural products comprised a wide range of compound classes, including flavonoids, alkaloids, lignans, anthraquinones, other diverse phenolics, and triterpenoids. The plant natural products were applied in two concentrations, namely 5 and 50 µM to the cell culture medium (Fig. 1a–h; Fig. S2; Fig. S3a–i).

Primary astrocytes were kept in culture under standardized conditions and 500 nM of oligomeric Aβ3(pE)-42 (Fig. S1) was added to the medium. Uptake of Aβ3(pE)-42 was monitored following fixation and immunostaining with corresponding antibodies (Fig. 1a, b). Quantitation of Aβ3(pE)-42 immunofluorescence was done double-blind with the GFAP mask as ROI (Fig. 1d). Although GFAP, a cytoskeletal marker, does not outline the complete cell volume of an astrocyte, previous work has shown that this measurement yields reliable data on the intracellular accumulation of Aβ3(pE)-42, both for smaller and bigger inclusions [19]. We first analyzed the total area of Aβ3(pE)-42 deposits (Fig. 1e). Out of 61 screened substances, nine significantly reduced the uptake of oligomeric Aβ3(pE)-42 to astrocytes (quercetin—50 µM *p* = 0.0002; rutin—5 µM *p* = 0.015; epicatechin—50 µM *p* = 0.0008; apigenin—5 µM *p* = 0.025; spinosin C—50 µM *p* = 0.002; formononetin—50 µM *p* < 0.0001; aristolactam BII—50 µM *p* < 0.0001; resveratrol—50 µM *p* = 0.0002; isoasarone—50 µM *p* = 0.003), while the others showed no major effect on Aβ3(pE)-42 internalization (Fig. 1e, Fig. S2, Fig. S3a, d, g). Correspondingly, these nine substances also decreased the number of discrete intracellular Aβ3(pE)-42 deposits (Fig. 1f), while for other plant substances, a reduced number was compensated by larger intracellular deposits (Fig. 1g, Fig. S2, Fig. S3c, f, i), thus keeping Aβ3(pE)-42 uptake unaltered. While the majority of substances yielded stronger effect at the 50 µM, some proved more efficient at 5 µM concentration which can be accounted for by different dose–response curves or additional, detrimental activities at very high concentrations. The strongest effect was observed for three substances: the flavonoid quercetin (3′,4′,5,7-tetrahydroxyflavon-3-ol), the flavonoid formononetin (7-hydroxy-3-(4-methoxyphenyl)-4H-chromen-4-one), and the alkaloid aristolactam BII (1,2-dimethoxydibenzo[*cd,f*]indol-4(5*H*)-one) (Fig. 1e–h). We selected two substances representing different classes of natural products for more detailed studies, the alkaloid aristolactam BII and the flavonoid quercetin. While protective effects of both flavonoids were previously recognized in AD [33, 34], the literature search revealed that only for quercetin it was demonstrated that it mediates reduction of Aβ secretion in mammalian cells and protects age-associated neurodegeneration in vivo [35]. Therefore, we decided to verify whether our screen reveals also a novel, protective function of this substance. Of note, the glycosylated form of quercetin, rutin, to a smaller extent, also inhibited Aβ3(pE)-42 uptake [36] (Fig. 1e–h).

**Fig. 2:**
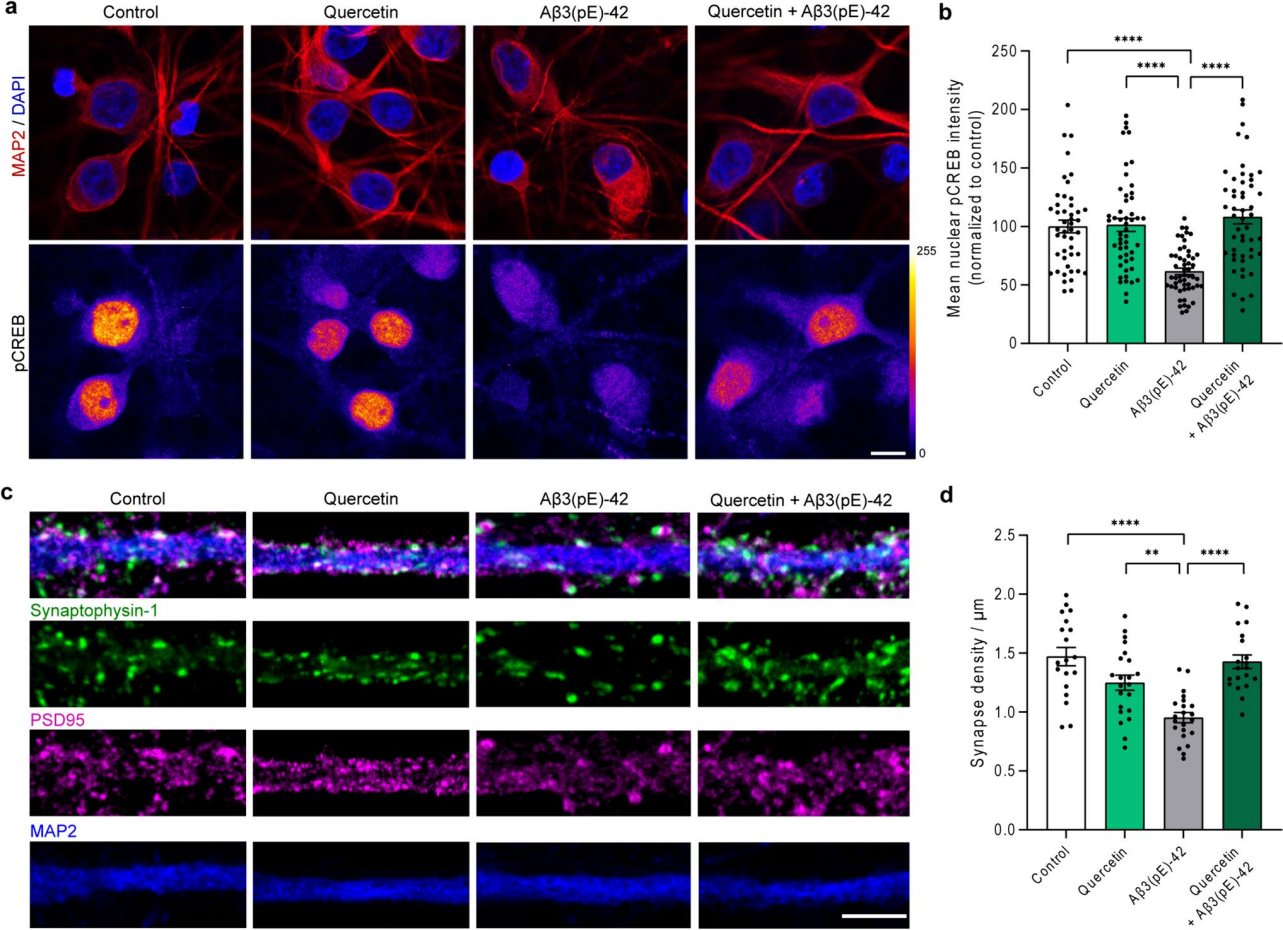
Quercetin rescues Aβ3(pE)-42-induced CREB shutoff and loss of synapse density in mixed hippocampal cultures. **a**, **b** Co-application of quercetin prevents Aβ3(pE)-42-induced loss in CREB Ser133 phosphorylation (CREB shutoff). **a** Representative confocal images of DIV18 mixed, neuronal-astrocytic cultures treated with 50 μM quercetin, 500 nM Aβ3(pE)-42 oligomers or 50 μM quercetin, and 500 nM Aβ3(pE)-42 oligomers for 72 h. Neurons were stained with MAP2 and pCREB antibodies and co-stained with DAPI. Scale bar is 10 μm. Lookup table indicates the pixel intensities from 0 to 255. **b** Mean pCREB fluorescence intensity within nuclear region (defined by DAPI) normalized to control. *N* = 46–53 nuclei from two independent cell cultures. **c**, **d** Co-application of quercetin prevents Aβ3(pE)-42-induced synapse loss. **c** Representative, confocal images of DIV18 mixed, neuronal-astrocytic cultures treated with 50 μM quercetin, 500 nM Aβ3(pE)-42 oligomers or 50 μM quercetin, and 500 nM Aβ3(pE)-42 oligomers for 48 h. Neurons were stained with Synaptophysin-1, PSD95, and MAP2 antibodies. Individual synapse was defined as overlapping or opposing Synaptophysin-1 and PSD95 immunosignal. Scale bar is 5 μm. **d** Mean number of synapses per 1 μm. *N* = 19–22 dendritic segments from at least two independent cultures. **b**, **d** **p < 0.01, ****p < 0.0001 by two-way ANOVA with post hoc Tukey test. Data are presented as mean $\pm$ s.e.m

Aβ3(pE)-42 is not taken up by neurons and does not associate with neuronal membranes [19]. Its neurotoxic effects are a consequence of astrocytic uptake, subsequent astroglial activation, and release of synaptotoxic, proinflammatory TNFα [18, 19]. Therefore, we next used primary, mixed, astrocytic-neuronal cultures to further characterize quercetin and aristolactam BII. A hallmark of amyloid-pathology in AD is decreased phosphorylation of neuronal transcription factor cAMP response element-binding protein (CREB), leading to its transcriptional inactivation termed CREB shutoff [37]. Transcriptional inactivation of neuronal CREB leads to the abolishment of pro-survival gene expression and hence the activation of pro-apoptotic signaling [38]. We therefore assessed CREB shutoff in mixed hippocampal cultures with a pCREB-specific antibody detecting Ser133 phosphorylated CREB (Fig. 2a, b). As reported previously, the addition of 500 nM Aβ3(pE)-42 to these cultures resulted in robust neuronal CREB shutoff [19]. Interestingly, however, co-application of quercetin to the medium attenuated CREB shutoff (Fig. 2a, b). Thus, quercetin appears to be effective to reduce measures of early neuronal dysfunction in primary cell cultures. The second candidate effectively blocking Aβ3(pE)-42 accumulation in astrocyte, aristolactam BII, did not rescue Aβ3(pE)-42-induced CREB shutoff (Fig. S4a, b). Therefore, we focused on quercetin as the most promising candidate to efficiently block Aβ3(pE)-42 uptake (Fig. 1, Fig. S5). Interestingly, we found that the substance was effective in preventing uptake of Aβ3(pE)-42 to HEK293T cells (Fig. S6a–d), pointing to a general role in preventing cellular accumulation of this causative agent for AD pathology.

The dephosphorylation of CREB at a crucial serine at position 133 (Fig. 2a, b) is tightly linked to Aβ-induced early synaptic dysfunction and synapse loss [37]. Alterations in synaptic structure and number can be observed relatively early and were also reported in in vitro AD models using mixed hippocampal cell cultures [4, 19] and we have already demonstrated that Aβ3(pE)-42-induced, proinflammatory signaling is synaptotoxic [18, 19]. We followed up on this observation and quantified synapse density in hippocampal primary neurons at DIV16-18 (Fig. 2c, d). A single synapse was defined as overlapping or opposing immunosignals of Synaptophysin-1 and PSD95 (pre-and postsynaptic marker respectively; Fig. 2c, d). In accord to a previous study, we found clear synapse loss in response to Aβ3(pE)-42 application to the cell culture medium [19]. Like in previous work, this loss was visible after 72 h and we have shown that it is induced by astroglial release of TNFα [19]. In support of a role in blocking Aβ3(pE)-42 astroglial uptake, co-application of quercetin to mixed hippocampal cultures significantly reduced this synapse loss (Fig. 2c, d).

**Fig. 3:**
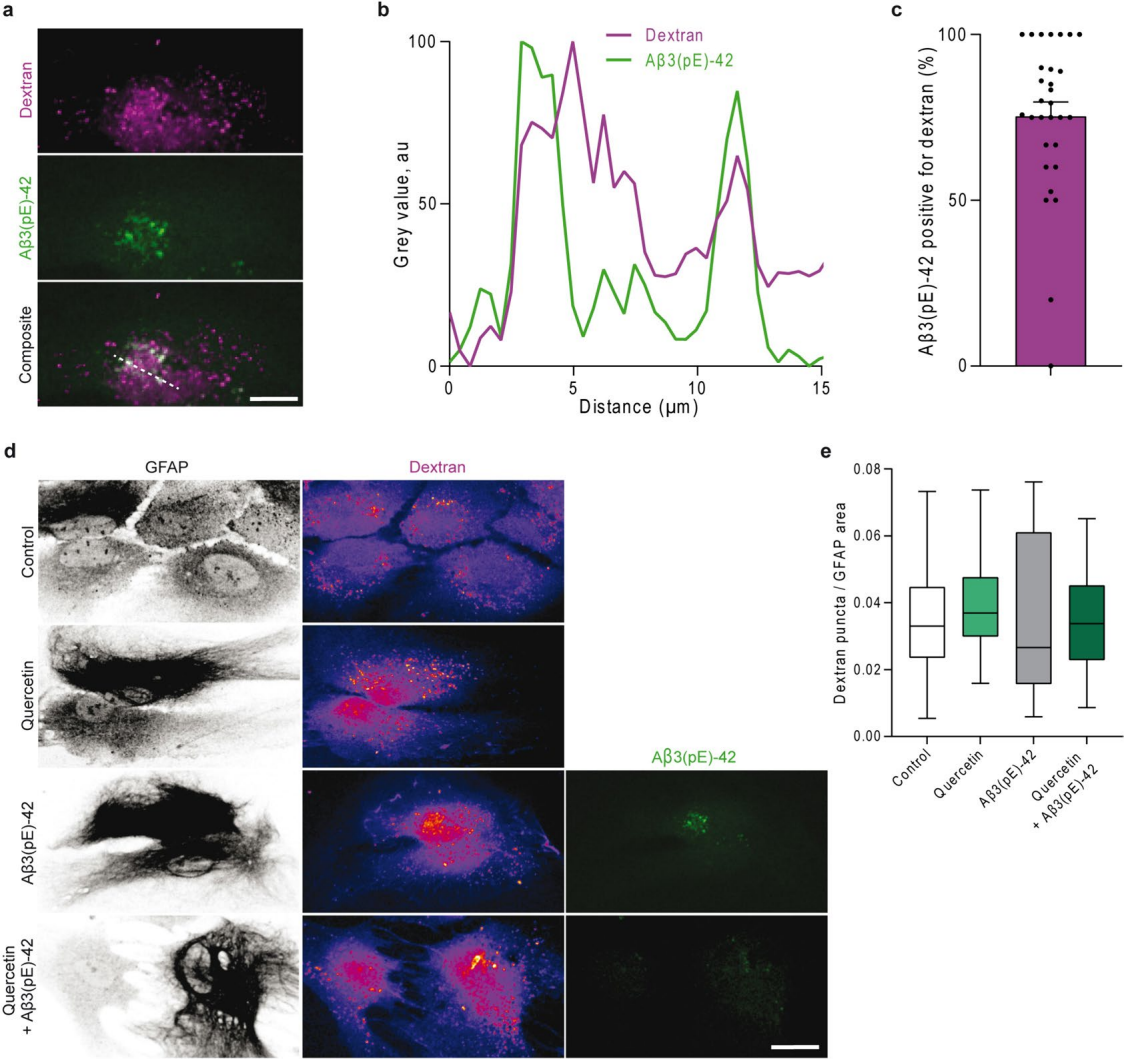
Quercetin prevents Aβ3(pE)-42 endocytosis. **a**–**c** Co-application of TMR-dextran and Aβ3(pE)-42 revealed prominent Aβ3(pE)-42 localization to the astrocytic endolysosomal system. **a** Representative, confocal images of primary astrocytes incubated with 5 mM TMR-dextran and 500 nM Aβ3(pE)-42. The dashed white line indicates the region along which the line profile was created. Scale bar is 10 µm. **b** The line profile of normalized mean grey values from the dextran and Aβ3(pE)-42 channel shows significant localization of Aβ3(pE)-42 to dextran-labeled endolysosomes. **c** Mean number of Aβ3(pE)-42 puncta (as % of total Aβ3(pE)-42 puncta within one cell) positive for TMR-dextran. *N* = 30 cells from two independent cultures. **d**, **e** Quercetin and Aβ3(pE)-42 do not influence general astrocytic endocytosis rate. **d** Representative, confocal images of primary astrocytes incubated with TMR-dextran with 50 µM quercetin, 500 nM Aβ3(pE)-42, or 500 nM Aβ3(pE)-42 + 50 µM quercetin. Scale bar is 30 µm. The image of the cell in **a** is a zoomed image of the Aβ3(pE)-42-treated group. **e** Box-whisker plot of the number of dextran puncta normalized to astrocytic area (defined by GFAP). Whiskers denote minimum and maximum value. Median is indicated within bar. *N* = 30–71 cells from two independent cell cultures. Ns for *p* > 0.05 by Kruskal–Wallis test followed by Dunn’s multiple comparisons test

The earliest phase of AD, often called “cellular” phase, is characterized by gradual alterations in neuronal and glial cells [1, 4, 6, 7, 39]. To verify, whether Aβ3(pE)-42 is endocytosed by astrocytes, we treated astrocytes with dynasore, a potent endocytosis inhibitor [40]. The co-application with dynasore revealed significant decrease in Aβ3(pE)-42 area, number of deposits, and the mean size of Aβ3(pE)-42 deposits (Fig. S7a–d). Next, we used TMR-dextran, an endolysosomal fluorescent marker [41]. We incubated dextran or dextran with Aβ3(pE)-42 for 2 h, followed by 2 h chase (Fig. 3a–c, Fig. S7e). Our data revealed that the vast majority (75%) of Aβ3(pE)-42 puncta localized in the astrocytic endolysosomal compartment. Importantly, we observed that neither quercetin nor Aβ3(pE)-42 affect the overall endocytosis rate measured by number of dextran puncta normalized to the area of astrocytes (Fig. 3d, e). In accordance with previous results (Fig. 1, Fig. S5), we observed decreased amounts of intracellular Aβ3(pE)-42. All in all, the data indicate that quercetin selectively prevents Aβ3(pE)-42 endocytosis.

**Fig. 4:**
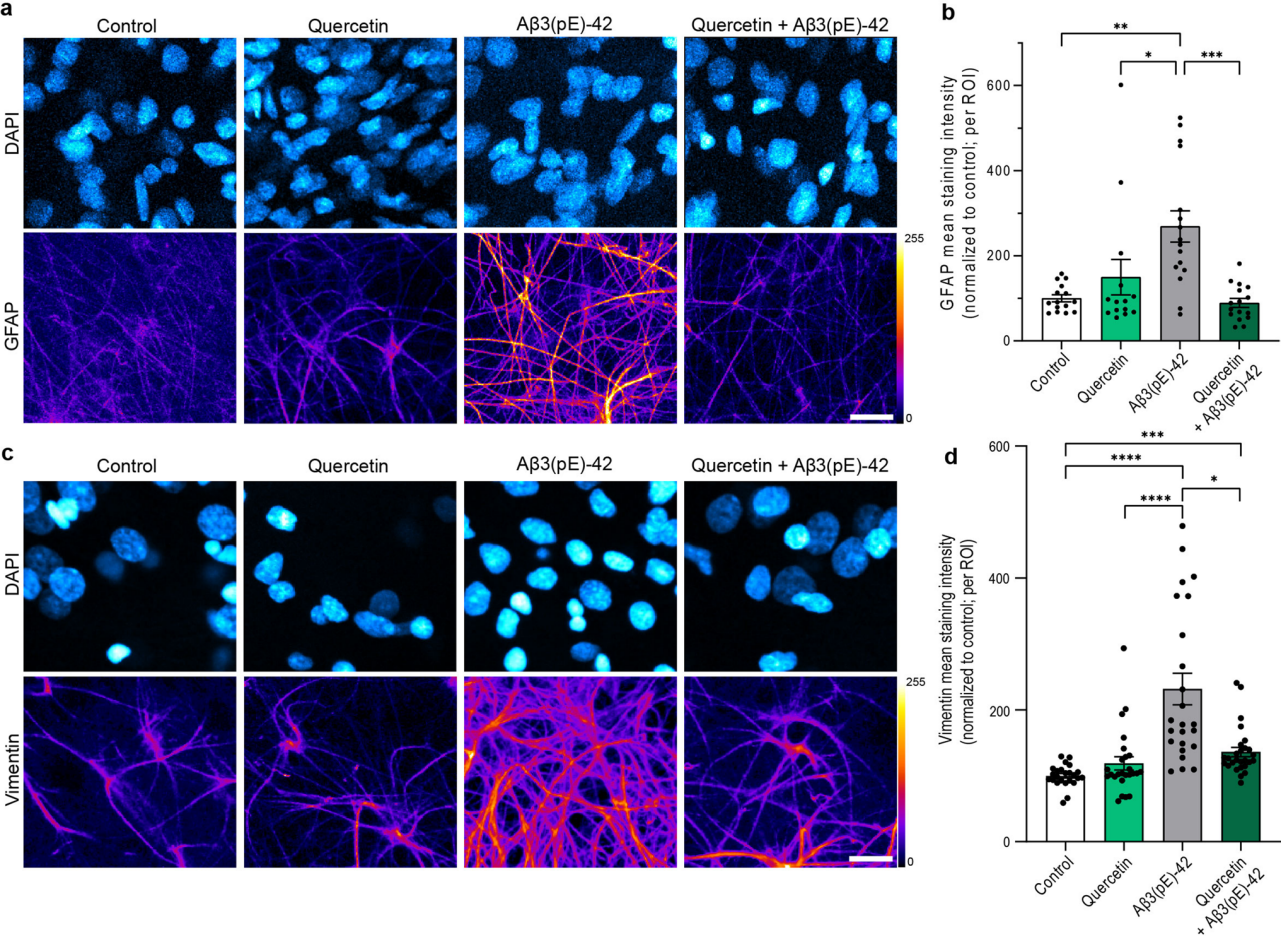
Quercetin prevents upregulation of GFAP and vimentin in astrocytes. **a**–**d** Co-application of quercetin prevents Aβ3(pE)-42-induced increased **a**, **b** GFAP or **c**, **d** vimentin immunoreactivity in organotypic hippocampal slices (OHSC). **a** Representative confocal images of DIV8 OHSC treated with 50 μM quercetin, 500 nM Aβ3(pE)-42 oligomers or 50 μM quercetin, and 500 nM Aβ3(pE)-42 oligomers for 24 h. OHSC were stained with antibody detecting GFAP and co-stained with DAPI (nuclear marker). Scale bar is 15 μm. Lookup table indicates the pixel intensities from 0 to 255. **b** Mean GFAP fluorescence intensity normalized to control. *N* = 14–16 ROIs from at least five slices per group from two independent cultures. **c** Representative confocal images of DIV8-15 OHSC treated with 50 μM quercetin, 500 nM Aβ3(pE)-42 oligomers or 50 μM quercetin, and 500 nM Aβ3(pE)-42 oligomers for 24 h. OHSC were stained with antibody detecting vimentin and co-stained with DAPI (nuclear marker). Scale bar is 15 μm. Lookup table indicates the pixel intensities from 0 to 255. **d** Mean vimentin fluorescence intensity normalized to control. *N* = 24–27 ROIs from at least three slices per group from two independent experiments. *p < 0.05, **p < 0.01, ***p < 0.001, ****p < 0.0001 by Kruskal–Wallis test followed by Dunn’s multiple comparisons test. Data are presented as mean $\pm$ s.e.m

Quercetin is a polyphenolic compound found in a variety of plants. It has many documented biological effects and a prominent anti-inflammatory action in a wide range of assays [33], including senolytic effects in senescent tissue [42, 43]. To test whether the protective effect of quercetin is associated with glia activation, we employed organotypic hippocampal slices, a three-dimensional culture system that preserves basic circuitry and cellular organization and that is therefore well suited to study glial proliferation as well as Aβ3(pE)-42 internalization. A common feature observed in AD is the transition of astrocytes to a state of reactive astrogliosis [6–8]. In previous work, it has been shown by us and others that oligomeric Aβ3(pE)-42 species induce a prominent glial inflammatory response that comes along with an increase in reactive GFAP-positive astroglia [6–8, 19]. Accordingly, we found that bath application of Aβ3(pE)-42 clearly increased GFAP-immunoreactivity in these slices (Fig. 4a, b), which is a measure of reactive astrogliosis [44]. When we co-administered quercetin at a concentration of 50 μM, the Aβ3(pE)-42-induced increase in GFAP-positive reactive astroglia was no longer visible (Fig. 4a, b). The same effect was observed for intermediate filament vimentin, another marker of reactive astrocytes [44] (Fig. 4c, d).

**Fig. 5:**
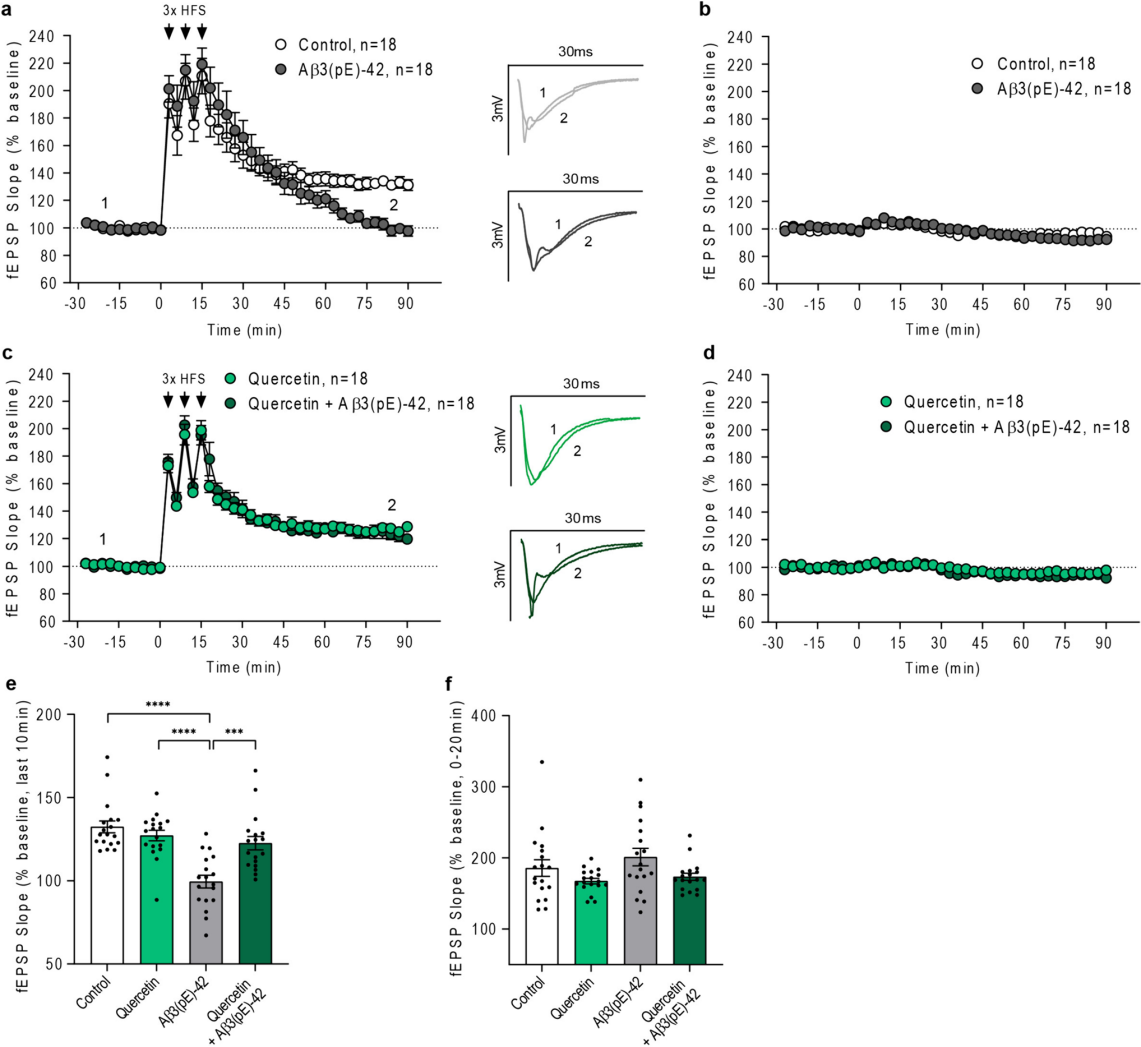
Quercetin rescues Aβ3(pE)-42-induced late LTP impairment. **a**–**d** LTP recordings revealed that **a** 2-h pre-treatment with Aβ3(pE)-42 oligomers causes impairment of late phase LTP, which is rescued by application of **c** quercetin, compared to control measurements. Accordingly, to the experimental group, the oligomers and quercetin were present in the bath during recordings. Insets show representative fEPSP analog traces at indicated time points: 1 = baseline, 2 = late LTP. **b**, **d** Basal synaptic transmission is not affected by bath application of **b** Aβ3(pE)-42 oligomers compared to a control and **d** by quercetin applied alone or with Aβ3(pE)-42 oligomers. **e**, **f** Dot plot representing **e** Aβ3(pE)-42-induced late phase LTP rescue by quercetin, while **f** early LTP and induction phase are not affected. *N* = 18 slices per group from at least six mice. **e**, **f** ***p < 0.001. ****p < 0.0001 by two-way ANOVA with Tukey’s multiple comparison test. Data are presented as mean $\pm$ s.e.m

Aβ3(pE)-42-induced glia activation leads to an impairment in synaptic function [19]. Therefore, to see whether quercetin can rescue synaptic impairment, we next moved to acute hippocampal slices. Long-term potentiation (LTP) at hippocampal Schaffer collateral synapses is considered to be a cellular model of learning and memory [45] and this type of synaptic plasticity is very early on interrupted by oligomeric Aβ [19, 28, 46, 47]. Field recordings revealed a severe impairment of the late phase of LTP, following 2-h pre-treatment and administration of oligomeric Aβ3(pE)-42 to the perfusion medium at a concentration of 500 nM (Fig. 5a, b). The reduced field excitatory postsynaptic potential (fEPSP) following induction of LTP is a common readout of the efficacy of oligomer preparation and therefore indicates that our preparation indeed resulted in the formation of synaptotoxic Aβ3(pE)-42 species like reported previously [19]. In line with a role of quercetin in the inhibition of oligomeric Aβ3(pE)-42 endocytosis in astrocytes, we observed a stunning rescue of late-phase LTP in slices co-treated with quercetin (Fig. 5c–f). The substance as such had no effect on the fEPSP during the time of recording (Fig. 5d).

## Discussion

Collectively, our data show that a simple screening approach can be used to identify bioactive substances extracted from plants that have neuroprotective effects based on the inhibition of oligomeric Aβ3(pE)-42 uptake to astrocytes. As proof of principle, we followed up on the neuroprotective effects of two hits from this first set of substances. While aristolactam BII did not prove efficient in preventing neuronal CREB shutoff, we identified a novel biological activity of quercetin—the prevention of Aβ3(pE)-42 astrocytic uptake, and, as a consequence, neuronal synaptic dysfunction and synapse loss.

Although it is well known that astrocytes endocytose neurodegeneration-associated proteins such as Aβ [7, 39, 48], the link between Aβ uptake, astrogliosis, and the disease onset and progression is unclear. The astrocytic phagocytic activity may result in neuroprotective reduction of Aβ concentration [49, 50]. On the other side, it was previously reported that the disruption of the astrocytic endolysosomal system is associated with AD progression [51, 52]. Among numerous posttranslationally modified Aβ species, Aβ3(pE)-42 is the most prominent one [53, 54]. Interestingly, unlike other Aβ forms, Aβ3(pE)-42 does not associate with neuronal membranes and is not taken up by neurons [19]. Instead, the oligomers are taken up by astrocytes, and accumulate in the endolysosomal system, which leads to its disruption and lysosomal membrane permeabilization [18, 19]. The intracellular leakage of lysosomal content induces upregulation of the proinflammatory signaling and release of TNFα, which drives Aβ3(pE)-42-induced synaptic stripping and LTP impairment [19]. This can be assigned to the unique biophysical properties of Aβ3(pE)-42, which displays higher propensity to aggregate and to form fibrillar species [55, 56]. Indeed, accumulation of fibrillar α-synuclein was reported to induce a lysosomal stress response characterized by loss of membrane integrity, disrupted catabolism and acidification, enlarged size, and increase in reactive oxygen species production [48, 57–59]. Thus, disruption of the endolysosomal system may result in a chronic inflammatory response that eventually leads to the release of proinflammatory cytokines and concomitant loss of physiological astrocytic functions, which in turn contribute to early synaptic dysfunction in AD [1, 60–63]. Very few approaches have centered on this process as a potential therapeutic target. We therefore aimed to identify plant-based substances with known biological activity that might interfere with Aβ3(pE)-42 uptake in astrocytes.

A long list of compounds deriving from herbal medicine have been in use or proposed for the treatment of neurological conditions, such as multiple forms of dementia, including AD [33, 64–68]. Plant-based substances are usually well tolerated and also well accepted as treatment options by the general public. This also applies to quercetin, which is a common component of food plants and even in clinical use for conditions of the heart and blood vessels and to prevent cancer [33, 69]. Its consumption is well tolerated and it has been safely used in doses up to 1 g daily for 12 weeks [69]. It has been previously reported that quercetin has antioxidant and anti-inflammatory effects [33, 70], which are probably its most prominent biological activities. Accordingly, quercetin has been shown to reduce neuronal cell death resulting from glutamate excitotoxicity [70, 71]. Recently, it has become the hallmark senolytic natural product for (synergistic) treatment of cancer and age-related diseases [33, 42, 43].

Furthermore, higher dietary intake of quercetin correlates with a slower rate of cognitive decline in healthy elderly individuals [72]. While the bioavailability of quercetin is low, and oral bioavailability and conversion of its glycones strongly depend on the gut microbiome [73, 74], the use of drug delivery systems, like nanosuspensions, can improve its oral adsorption [75]. Furthermore, its ability and rates to cross the blood–brain barrier require further investigation [33, 76, 77]. Likewise, the concentration-dependent effects on neuronal and glial cells warrant further studies. Some reports suggest that, depending on concentration and its metabolism, quercetin may exert the neuroprotective or neurotoxic function [77–79]. In this regard, the presence of glia in the in vitro system seems to be crucial, as glial metabolism of quercetin into glutathione conjugates leads to the reduction of neurotoxic effects of quercetin [80]. These findings also account for the fact that we did not observe any neurotoxic effects, in our mixed co-culture system containing both neurons and astrocytes or quercetin-dependent increase in neuronal CREB phosphorylation [78]. Along these lines, although quercetin acts as a phosphoinositide (PI) 3-kinase inhibitor which could potentially lead to the disruption in membrane trafficking [81–83], the dextran assay did not reveal any impairment in astrocytic endocytosis. Nevertheless, various neuroprotective effects have been reported for quercetin in various disease states including mouse models of AD [33, 72]. Interestingly, a recent study revealed that quercetin decreases the amount of secreted Aβ and rescued toxic effects of Aβ in vivo [35].

Here, using exogenously applied oligomers at defined concentration, we report a hitherto unknown biological activity that might become relevant in the context of amyloid-pathology in the brain. We found that quercetin efficiently blocked the endocytosis and accumulation of Aβ3(pE)-42 in astrocytes. At present, the mechanism by which quercetin exerts its effect on astrocytic Aβ3(pE)-42 accumulation is unclear. Since the dextran uptake assay revealed that quercetin has not significantly altered the overall endocytosis rate, a plausible explanation is that quercetin sequesters Aβ3(pE)-42 oligomers, thus preventing binding to astrocytic endocytic receptors [84]. It was shown that quercetin can bind to fibrillar α-synuclein and Aβ [85, 86]. Since pyroglutaminylation increases the hydrophobicity of soluble Aβ oligomers, this Aβ modification could facilitate binding to hydrophobic quercetin in a similar manner like to fibrillar species [87–89]. Another intriguing possibility concerns the regulation of ApoE expression and function. The APOEε4 gene is a genetic risk factor for LOAD, likely through its strong effect on the accumulation of Aβ [51, 90]. ApoE is mainly produced by astrocytes and astrocyte-derived ApoE is pivotal for clearance of oligomeric Aβ [51, 90, 91]. It was shown that quercetin increases ApoE levels most likely by inhibiting ApoE degradation [92]. While this might be an important aspect with respect to a long-term treatment, we here report an effect that is rather rapid and will occur before regulation of gene expression might kick in. Thus, the acute consequences of quercetin are likely a reduced uptake of Aβ3(pE)-42. Furthermore, other effects of long-term quercetin administration in vivo and in vitro on gene networks that were analyzed in silico and that might provide neuroprotection in the context of AD [33, 35, 93, 94] are unlikely to contribute to the acute effects of quercetin on the accumulation of Aβ3(pE)-42 in astrocytes.

Uptake of oligomeric Aβ has been shown for several different cell types [1, 8]. The mechanisms are to a large extent unclear and might in fact differ between neurons, microglia, and astrocytes [52]. Since quercetin also blocked uptake of Aβ3(pE)-42 in HEK293T cells, we speculate that the mechanism underlying its action is of general relevance. A direct interaction of quercetin with Aβ3(pE)-42 would explain the observed effects. Such a direct interaction was indeed recently reported for full-length Aβ [95]. Other studies have shown that OH groups and phenolic rings in flavonoids are essential for the noncovalent interactions with β-sheet structures, which are common to all amyloid proteins [96]. A consequence of quercetin binding to full length Aβ seems to be the prevention of fibril formation [95]. While it is unclear whether this also applies to Aβ3(pE)-42 quercetin binding, subsequent prevention of oligomerization cannot account for the effect of quercetin on Aβ3(pE)-42 accumulation in astrocytes since we have used preformed oligomers. However, if quercetin binds to oligomerized Aβ3(pE)-42, it is possible that this binding will hinder uptake.

Taken together, our study demonstrates that quercetin, by yet unknown mechanism, efficiently blocks astrocytic uptake of Aβ3(pE)-42 thus preventing Aβ3(pE)-42-induced synaptic impairment. In conjunction with other published work, these results point to a plausible scenario where this flavonoid is effective in preventing amyloid pathology by simultaneously influencing Aβ endocytosis, metabolism, and assembly of Aβ oligomeric species.

## Supplementary Information

Below is the link to the electronic supplementary material.

Supplementary file1 (DOCX 3724 KB)

## Data Availability

The original contributions presented in the study are included in the article/Supplementary Material. The datasets and material used in the study are available from corresponding authors on reasonable request.
