# Supplementary material for: A Screen of Plant-Based Natural Products Revealed That Quercetin Prevents Pyroglutamylated Amyloid-β (Aβ3(pE)-42) Uptake in Astrocytes As Well As Resulting Astrogliosis and Synaptic Dysfunction
Source: Mol Neurobiol. 2024 Sep 25;62(3):3730–45. doi: 10.1007/s12035-024-04509-6 (PMC11790700; doi:10.1007/s12035-024-04509-6)
Supplement: Supplementary file 1 — Supplementary file1 (DOCX 3724 KB) [file 12035_2024_4509_MOESM1_ESM.docx]

Supplementary Information

A screen of plant-based natural products revealed that quercetin prevents amyloid-β uptake in astrocytes as well as resulting astrogliosis and synaptic dysfunction

Journal: Molecular Neurobiology

Helene Arndt^1^, Mark Bachurski^1^, PingAn Yuanxiang^1^, Katrin Franke^2,3,4^, Ludger A. Wessjohann^2,4,5^, Michael R. Kreutz^1, 6, 7,8*^, Katarzyna M. Grochowska^1,6*^

^1^Research Group Neuroplasticity, Leibniz Institute for Neurobiology, 39118 Magdeburg, Germany

^2^Department of Bioorganic Chemistry, Leibniz Institute of Plant Biochemistry, 06108 Halle, Germany

^3^Institute of Biology/Geobotany and Botanical Garden, Martin Luther University Halle-Wittenberg, 06108 Halle, Germany

^4^German Centre for Integrative Biodiversity Research (iDiv) Halle-Jena-Leipzig, 04103 Leipzig, Germany

^5^Institut für Chemie, Chair of Natural Products Chemistry, Martin-Luther-University Halle-Wittenberg, 06120 Halle (Saale)

^6^Leibniz Group ‘Dendritic Organelles and Synaptic Function’, Center for Molecular Neurobiology, ZMNH, University Medical Center Hamburg-Eppendorf, 20251 Hamburg, Germany

^7^German Center for Neurodegenerative Diseases (DZNE), 39120 Magdeburg, Germany

^8^Center for Behavioral Brain Sciences, Otto von Guericke University, 39120 Magdeburg, Germany

*** Correspondence:**Michael R. Kreutz: Michael.Kreutz@lin-magdeburg.de; ORCID ID: 0000-0003-0575-6950
Katarzyna M. Grochowska: Katarzyna.Grochowska@zmnh.uni-hamburg.de; ORCID ID: <https://orcid.org/0000-0001-8298-176X>


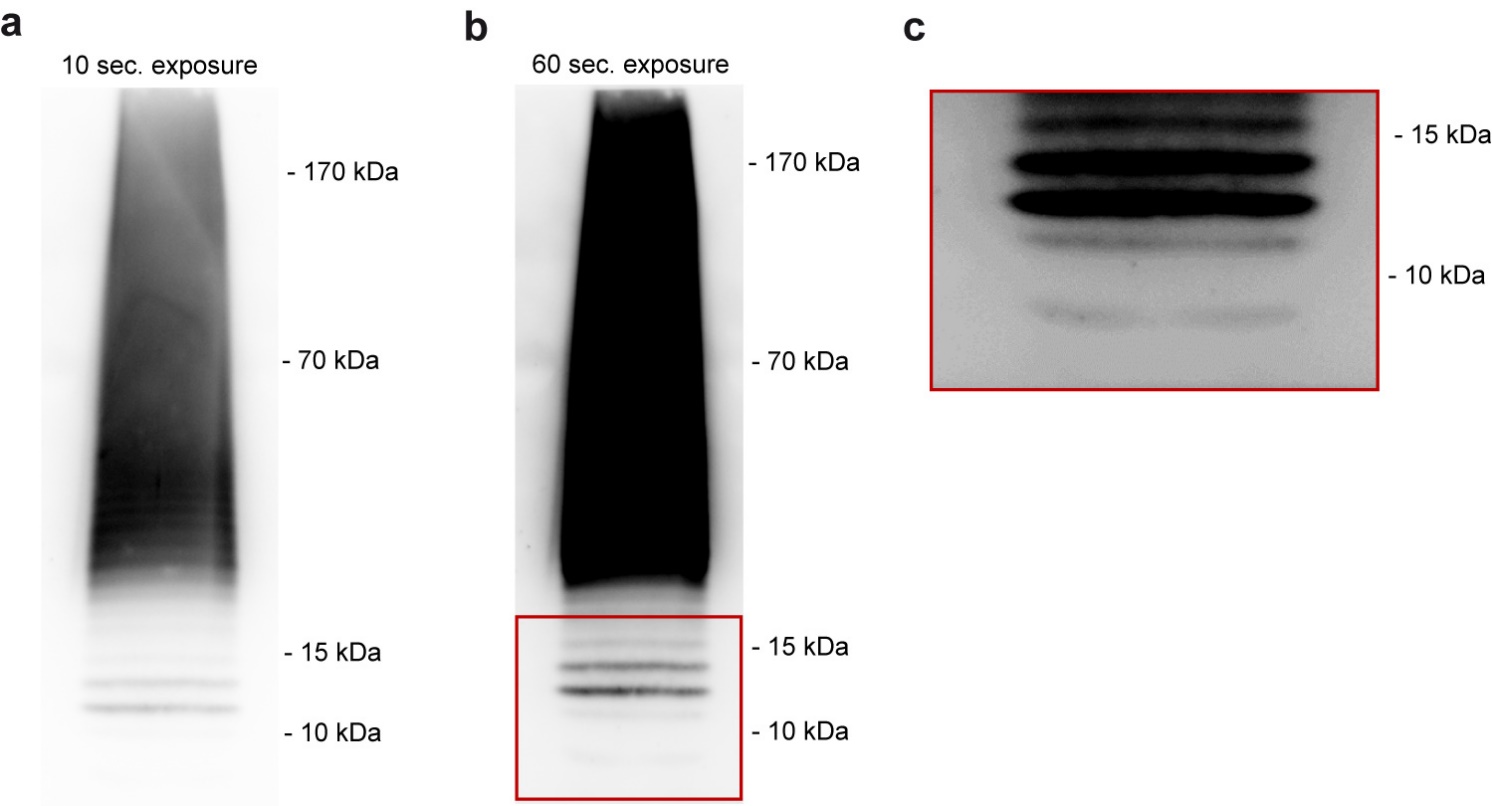


Supplementary Fig. 1 Characterization of oligomeric Aβ3(pE)-42 preparation

**(a-c)** SDS-PAGE of Aβ(pE)-42 oligomeric preparation showed protein sizes from di- and trimeric oligomers (8 and 12 kDa) up to larger oligomeric protein complexes (>170 kDa). Depicted are two Western blot exposure times of **(a)** 10 sec. and **(b, c)** 60 sec. **(c)** is an enlarged region of interest of **(b,** denoted by red box). The linear brightness and contrast was applied to enhance the visibility of the oligomeric species of lower molecular weight.

Supplementary Fig. 2 Structures of tested substances

Green color indicates substances that significantly reduced the uptake of oligomeric Aβ3(pE)-42 to astrocytes.


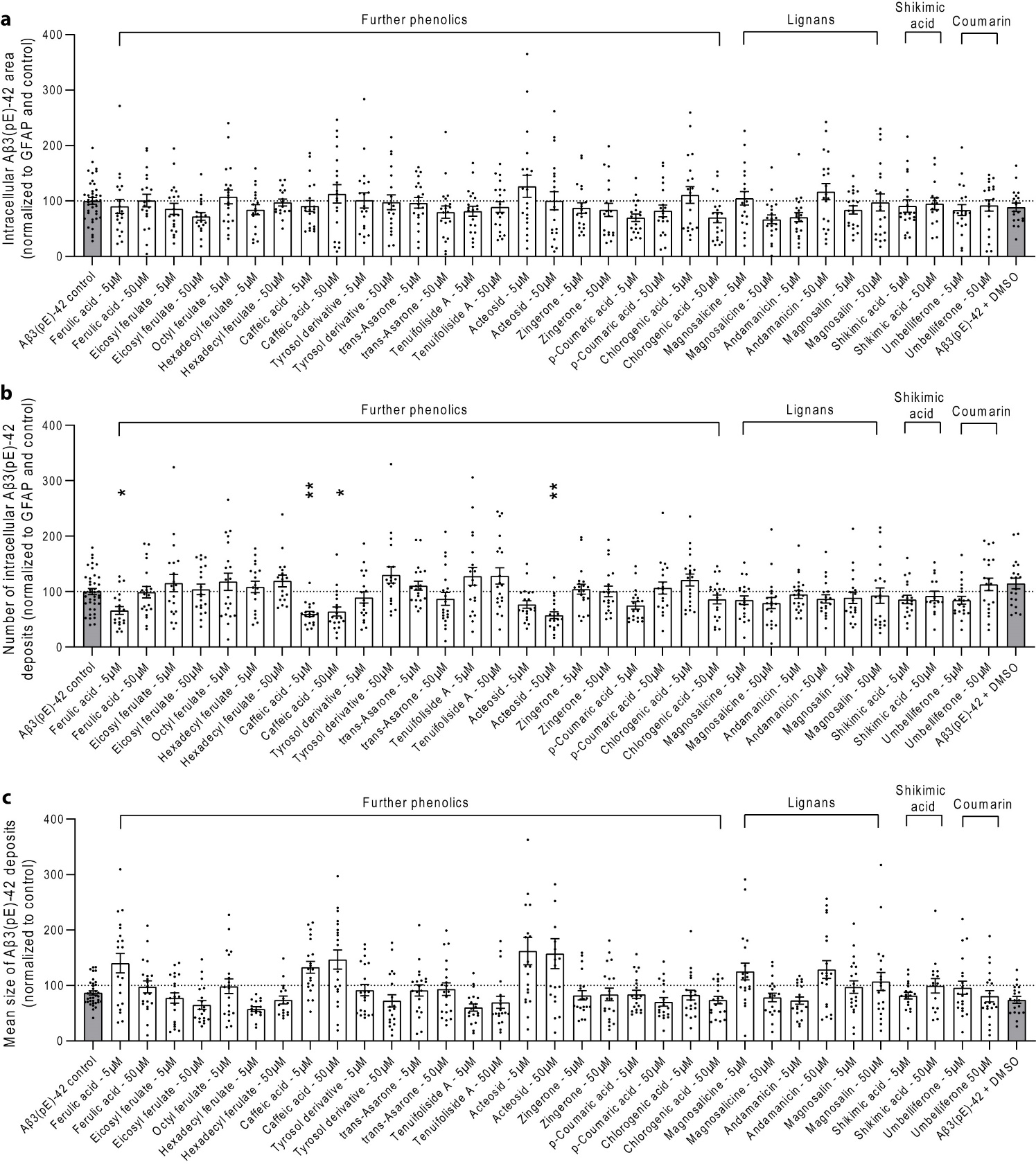


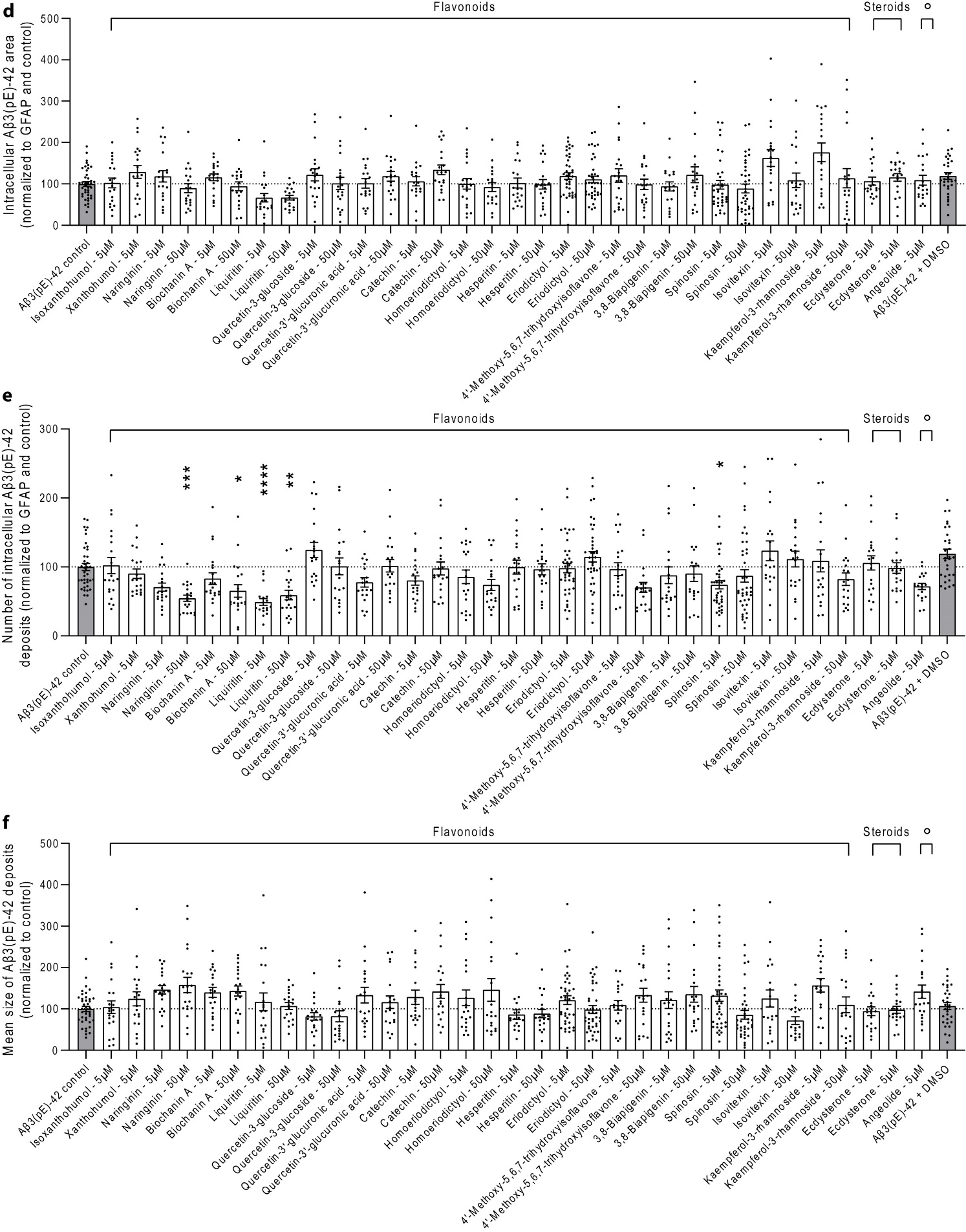

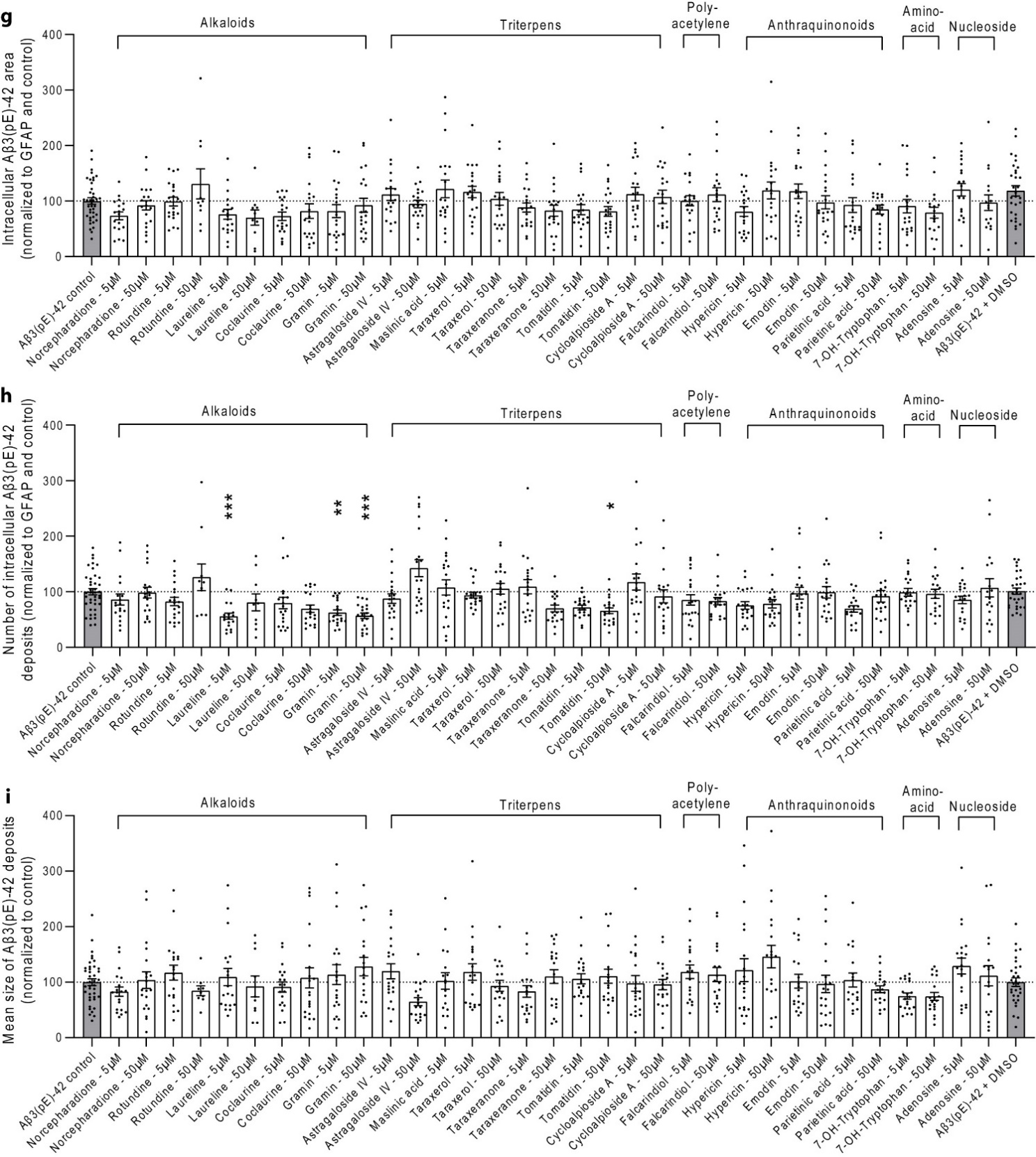


**Supplementary Fig. 3 Plant substances that showed no major significant alterations regarding uptake of intracellular Aβ3(pE)-42 deposits in astrocyte cultures at the concentrations tested**

**(a-i)** Control (incubation with Aβ3(pE)-42 only) and Aβ3(pE)-42 plus plant solvent DMSO are highlighted in grey. ° = Butylidenephthalide; Norcepharadione = Norcepharadione B; Taraxeranone = 14a-Taraxeran-3-one; 7-OH-Tryptophan = 7-Hydroxy-tryptophan; Biochanin A = 5,7-Dihydroxy -4‘-methoxy-isoflavone; Ecdysterone = 20-Hydroxyecdysone; Isovitexin = Isovitexin-2''-O-b-D-glucopyranosid. *p<0.05, **p<0.01 by Kruskal-Wallis test followed by Dunn’s multiple comparisons test. Data are presented as mean $\pm$ s.e.m.


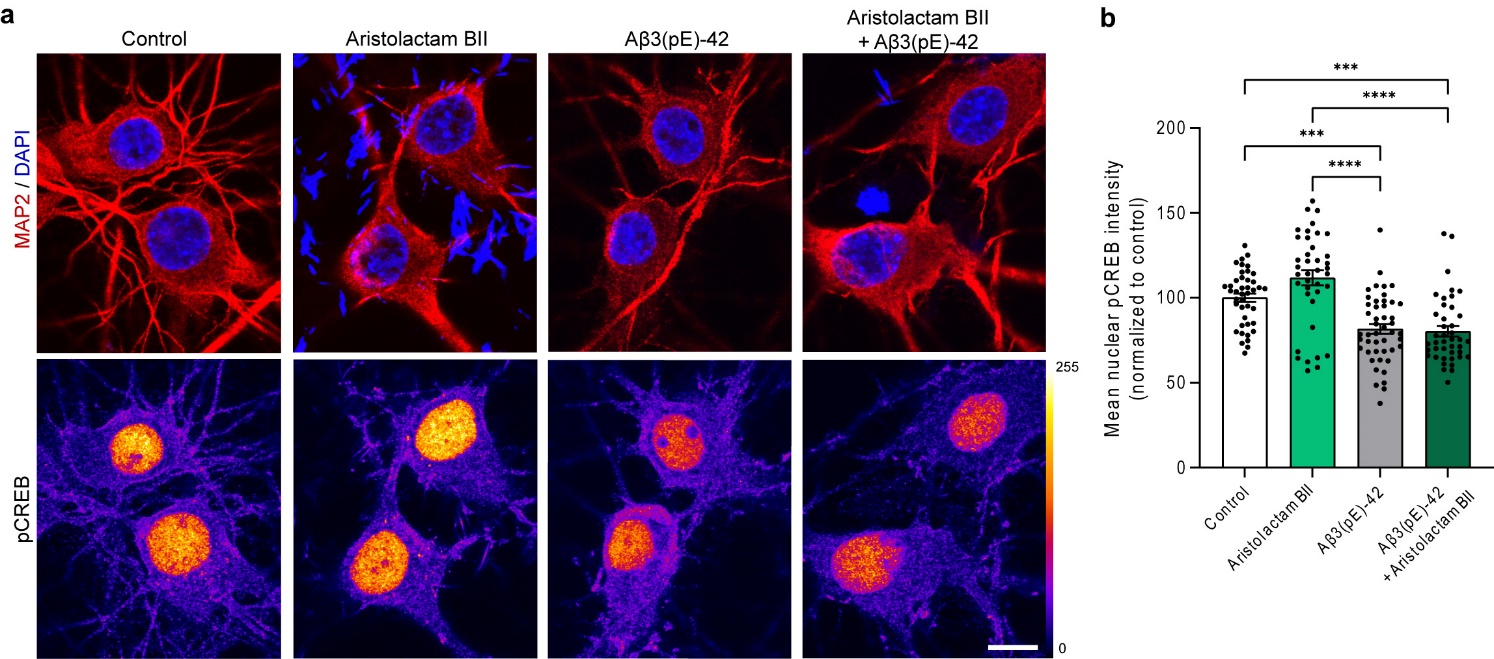


**Supplementary Fig. 4 Aristolactam BII does not rescue Aβ3(pE)-42-induced CREB shutoff**

**(a)** Representative confocal microscopy images of DIV18 neurons treated with 50 μM aristolactam BII, 500 nM Aβ3(pE)-42 oligomers or 50 μM aristolactam BII and 500 nM Aβ3(pE)-42 oligomers for 72h. Neurons were stained with MAP2 and pCREB antibodies and co-stained with DAPI. Scale bar is 10 μm. Lookup table indicates the pixel intensities from 0 to 255. **(b)** Mean pCREB fluorescence intensity within nuclear region (defined by DAPI) normalized to control. N= 39-47 nuclei from 2 independent cell cultures. ***p<0.001, ****p<0.0001 by Kruskal-Wallis followed by Dunn’s multiple comparisons test. Data are presented as mean $\pm$ s.e.m.


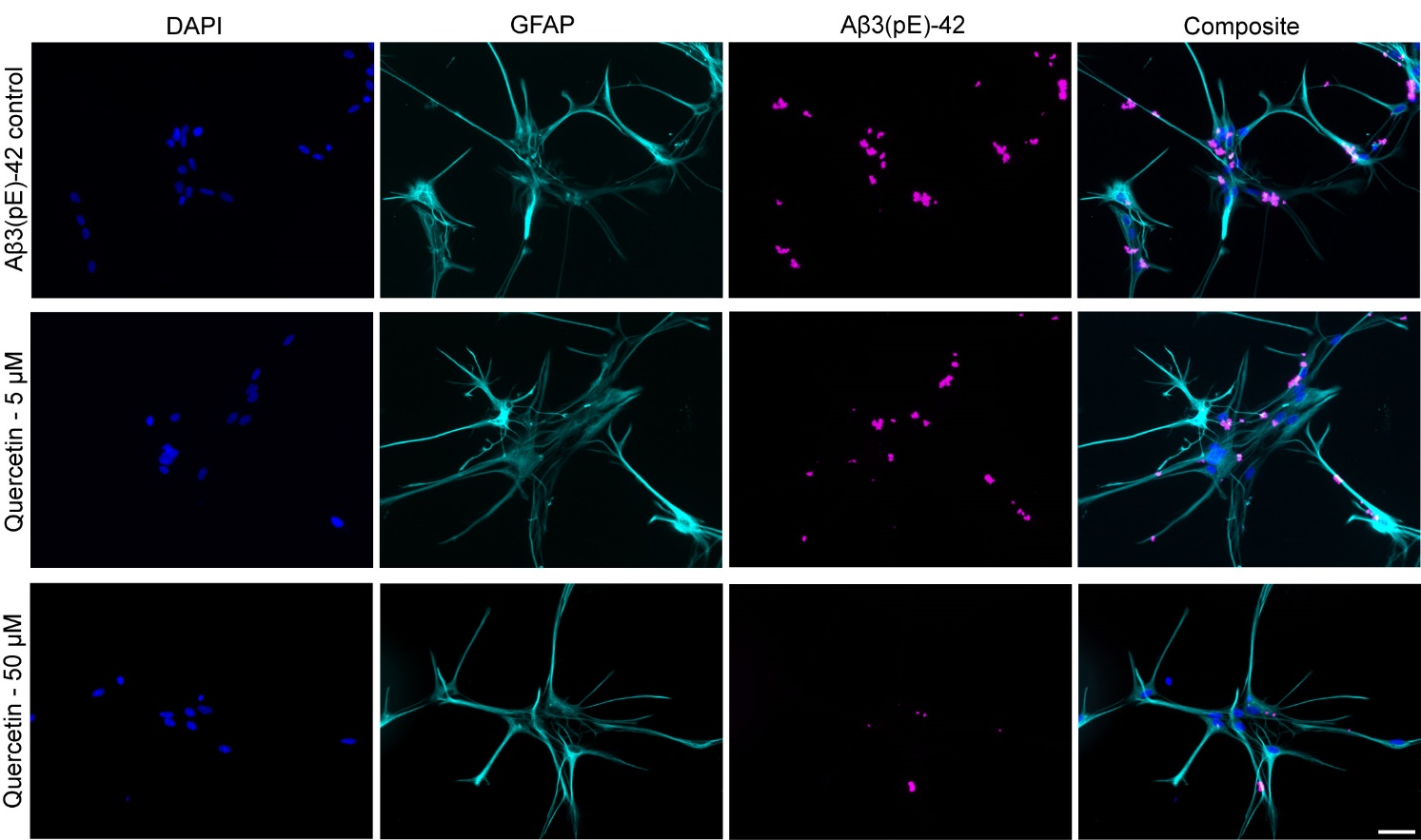


**Supplementary Fig. 5 Representative fluorescence microscopy images of primary astrocytes incubated with 500 nM Aβ3(pE)-42 only or 500 nM Aβ3(pE)-42 plus 5 µM or 50 µM quercetin for 16h.**

Astrocytes were stained with GFAP and Aβ3(pE)-42 antibodies and co-stained with DAPI. Scale bar is 50 µm.


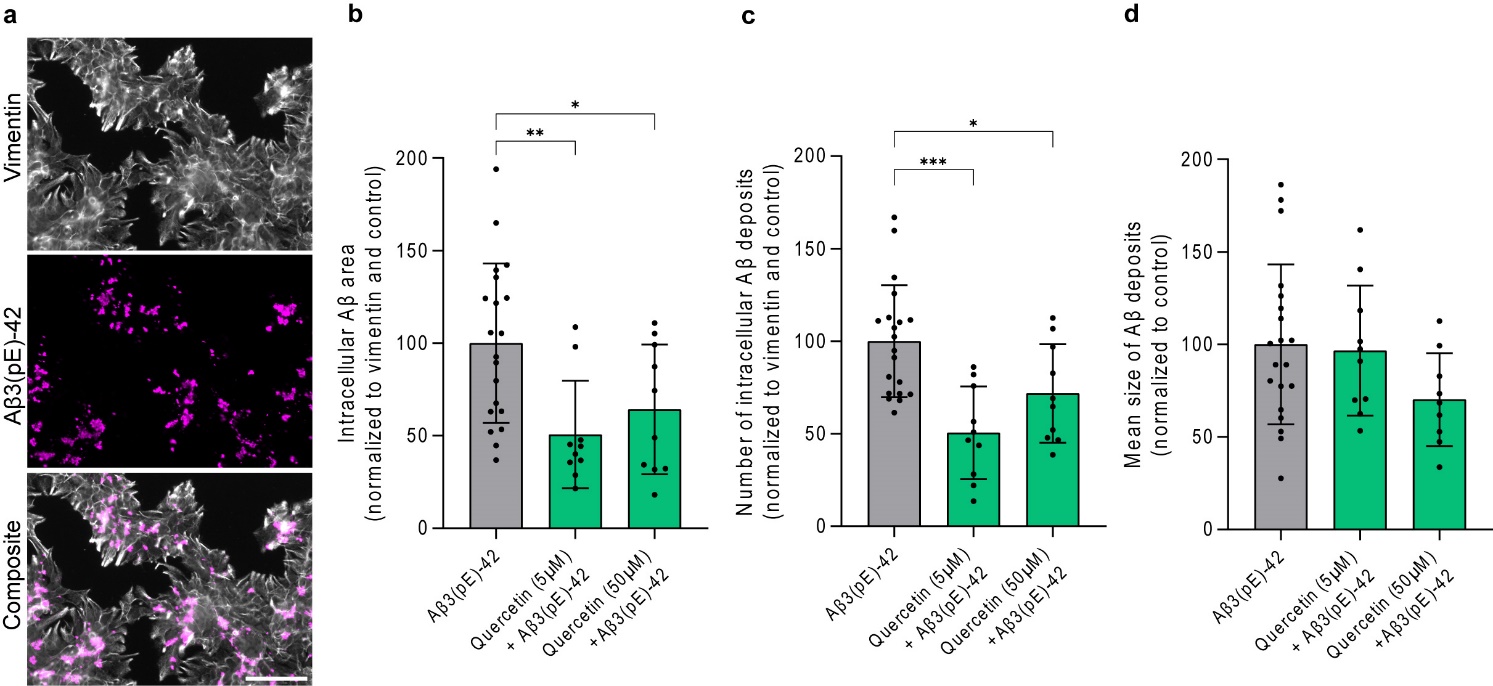


**Supplementary Fig. 6 Quercetin reduces uptake and number of intracellular Aβ3(pE)-42 deposits in HEK-293T cell cultures**

**(a)** Representative fluorescence microscopy images of HEK-293T cells treated for 10h with Aβ3(pE)-42 and stained with antibodies detecting Aβ3(pE)-42 and vimentin. Scale bar is 100 μm. **(b-d)** Treatment with both 5 μM and 50 μM quercetin reduces **(b)** area and **(c)** number but not **(d)** mean size of intracellular Aβ3(pE)-42 deposits. N = 10 FOVs. *p<0.05, **p<0.01, ***p<0.001 by one-way ANOVA followed by Dunnett’s multiple comparison test. Data are presented as mean $\pm$ s.e.m.

**
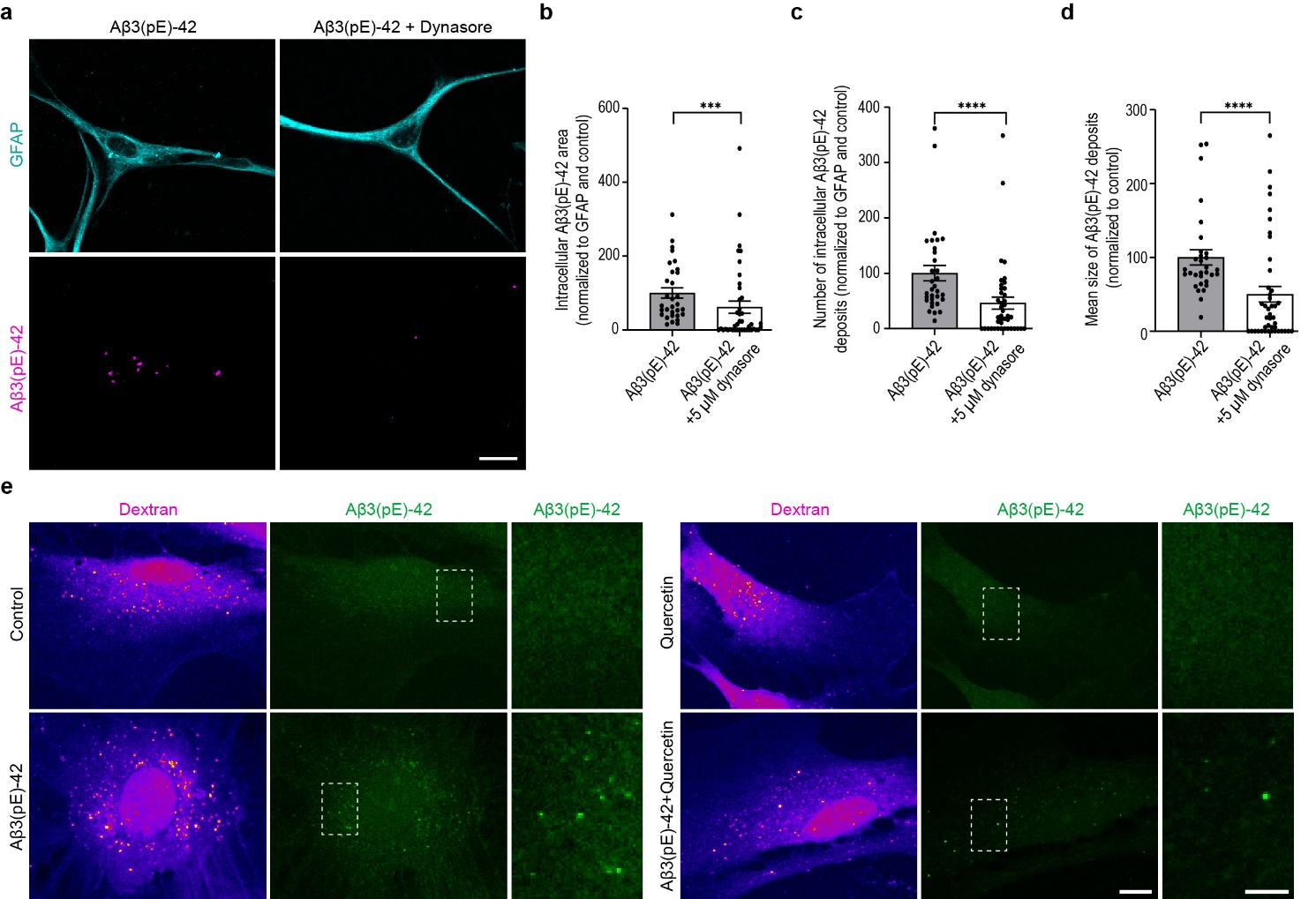
Supplementary Fig. 7 Quercetin reduces Aβ3(pE)-42 deposits endocytosis.**

**(a)** Representative fluorescence microscopy images of primary astrocytes treated for 16h with Aβ3(pE)-42 and dynasore. stained with antibodies detecting Aβ3(pE)-42 and GFAP. Scale bar is 20 μm. **(b-d)** Treatment with both 5 μM dynasore reduces **(b)** area, **(c)** and **(d)** mean size of intracellular Aβ3(pE)-42 deposits. N = 31-41 cells from 2 independent experiments. ***p<0.001, ****p<0.0001 by Mann-Whitney test. Data are presented as mean $\pm$ s.e.m. **(e)** There is no Aβ3(pE)-42 immunosignal in astrocytes not treated with Aβ3(pE)-42. Representative fluorescence microscopy images of primary astrocytes incubated with TMR-dextran with 50 µM quercetin, 500 nM Aβ3(pE)-42, or 500 nM Aβ3(pE)-42+50 µM quercetin. Scale bar is 10 µm. White, dashed line marks the enlarged region. The inset scale bar is 5 µm.
